# The role of protein-protein interactions mediated by the PB1 domain of NLP transcription factors in nitrate-inducible gene expression

**DOI:** 10.1186/s12870-019-1692-3

**Published:** 2019-02-28

**Authors:** Mineko Konishi, Shuichi Yanagisawa

**Affiliations:** Biotechnology Research Center, The University of Tokyo, Yayoi 1-1-1, Bunkyo-ku, Tokyo, 113-8657 Japan

**Keywords:** NIN-LIKE PROTEIN (NLP), Nitrate response, PB1 domain, Protein-protein interaction

## Abstract

**Background:**

NIN-LIKE PROTEIN (NLP) transcription factors are master regulators of nitrate-inducible gene expression in higher plants. NLP transcription factors contain a nitrate signal-responsive domain in the amino-terminal region, an RWP-RK-type DNA-binding domain in the middle, and a Phox and Bem1 (PB1) domain at the carboxy terminus. Although the PB1 domain of NLP transcription factors appears to mediate protein-protein interactions associated with nitrate-inducible gene expression in higher plants, its precise role in nitrate-inducible gene expression has not previously been characterized.

**Results:**

Yeast two-hybrid assays with the PB1 domain of the Arabidopsis transcription factor NLP7 revealed NLP-NLP interactions that required the core amino acid residues (K867, D909, D911, and E913) within the PB1 domain. Consistent with previous speculation on redundant and overlapping functions between different Arabidopsis NLP transcription factors, NLP-NLP interactions were observed between a variety of combinations of different NLP transcription factors. Furthermore, a mutated form of NLP7 that harbored amino acid substitutions at K867, D909, D911, and E913 required a far higher level of expression than wild-type NLP7 to restore nitrate-responsive gene expression and growth of *nlp6 nlp7–1* double mutants. Surprisingly, however, the ability to transactivate nitrate-responsive promoters in protoplast transient expression assays was similar between wild-type and mutant forms of NLP7, suggesting that the PB1 domain was not required for transcription from naked DNA.

**Conclusions:**

Protein-protein interactions mediated by the PB1 domain of NLP transcription factors are necessary for full induction of nitrate-dependent expression of target genes *in planta*. The PB1 domains of NLP transcription factors may act on gene expression from chromosomal DNA via homo- and hetero-oligomerization in the presence of nitrate.

**Electronic supplementary material:**

The online version of this article (10.1186/s12870-019-1692-3) contains supplementary material, which is available to authorized users.

## Background

Plants acquire nitrogen (N) from soils in the form of nitrate and ammonium ions, which are assimilated into amino acids and then used to generate N-containing organic molecules such as chlorophylls, nucleotides, and proteins. Nitrate is a major form of inorganic N in upland oxidative soils, where the majority of higher plant species are found, and nitrate supply is often a limiting factor for plant growth [1]. Nitrate ions are applied to soils as fertilizers or produced by nitrification activity of microorganisms but, due to their negatively charged nature, they do not remain on soil particles for long and thus easily leach out from soils [2]. The availability of nitrate to plants therefore fluctuates and is strongly influenced by rainfall, irrigation, and fertilizer application.

Plants have regulatory mechanisms that sense nitrate and/or nitrogen status and then modulate gene expression to fine-tune metabolism and growth in response to nitrate availability and internal nitrogen demand [3–8]. Nitrate is one of the key signaling molecules that orchestrates gene expression to optimize nitrate utilization and reconcile growth with nitrate availability [3, 9–12]. The molecular framework for nitrate-induced gene expression in higher plants has been elucidated in recent years. The latest model of this framework suggests that nitrate signals induce phosphorylation and activation of NIN-LIKE PROTEIN (NLP) transcription factors, with the help of group III calcium-dependent proteins kinases, including CPK10, CPK30, and CPK32 [13–17].

A single plant species may possess multiple genes encoding NLP transcription factors [17–20]; the Arabidopsis genome, for example, contains nine *NLP* genes (*NLP1–9*). The high similarities between the amino acid sequences of different NLP transcription factors suggest that they have redundant and overlapping functions in nitrate-inducible gene expression, although some NLP transcription factors may have somewhat different physiological roles. NLP7 appears to play a dominant role in Arabidopsis, as single mutations in *NLP7* cause significant decreases in the expression levels of some nitrate-inducible genes and reduce nitrate-dependent growth promotion [14, 21, 22]. Of the other *NLP* genes, *NLP6* resembles *NLP7* most closely, but disruption of *NLP6* alone does not cause obvious defects; however, mutation of *NLP6* in combination with the *nlp7–1* mutation exacerbates changes in gene expression and growth defects [23], demonstrating the redundant roles of NLP6 and NLP7. On the other hand, NLP8 regulated nitrate-promoted seed germination in Arabidopsis [24].

The physiological functions of NLP transcription factors have been analyzed using NLP6-SUPRD transgenic Arabidopsis plants, an approach that bypasses the problems caused by redundancy between *NLP* genes [25, 26]. NLP6-SUPRD is a chimeric repressor form of NLP6 that consists of NLP6 fused to a transcriptional repressor domain; it is highly expressed in NLP6-SUPRD plants and thus out-competes the endogenous NLP transcription factors. NLP6-SUPRD plants show severe growth inhibition when either nitrate or ammonium nitrate is the sole N source [13]. Moreover, expression of most of the nitrate-inducible genes involved in various cellular processes, including nitrate transport and assimilation, carbohydrate metabolism, and hormone responses, is also inhibited in NLP6-SUPRD plants [26]. Thus, NLP transcription factors likely coordinate anabolism and growth by acting as master regulators of nitrate-inducible gene expression. Consistent with the results obtained from *nlp7* mutants and NLP6-SUPRD plants, growth of Arabidopsis is improved by over-expression of native NLP7, and also by over-expression of *Zm*NLP6 or *Zm*NLP8 from maize [20, 27], indicating that NLP transcription factors modulate N usage in plants. Recently, it has also been reported that in legumes, some NLP transcription factors regulate nitrate-responsive gene expression for the promotion of nitrate uptake and assimilation as well as the modulation of nodulation [16, 17].

NLP transcription factors contain three well-conserved domains: a nitrate signal-responsive domain in the amino-terminal region, an RWP-RK-type DNA-binding domain in the middle, and a Phox and Bem1 (PB1) domain at the carboxy terminus [18, 26]. The nitrate signal-responsive domain mediates activation of NLP transcription factors in response to nitrate [13, 24]. The conserved serine residue (serine 205 in NLP7) within this region is phosphorylated by CPK10, CPK30, and CPK32 upon the perception of nitrate signals [15]. The RWP-RK-type DNA-binding domain, named because it contains the conserved amino acid sequence Arg-Trp-Pro-X-Arg-Lys (where X indicates any amino acid), binds to nitrate-responsive *cis*-elements (NREs), which were originally identified in the promoters of the Arabidopsis nitrite reductase gene *NIR1* and other nitrate-inducible genes [13, 28–30]. The RWP-RK DNA-binding domain alone can bind to NREs, independently of nitrate [13]. The PB1 domain is thought to be involved in protein-protein interactions, as described below. Guan et al. (2017) recently showed, using NLP6 and NLP7 from Arabidopsis, that the NLP-NLP interaction, and also the interaction between NLP and the TCP20 transcription factor, is mediated by the PB1 domain [23]. They also suggested, based on the interaction between NLP6/7 and TCP20 in nuclei and the effect of the *tcp20* mutation on expression of *NRT1.1*, *NIA1*, and *NIA2*, that the NLP6/7-TCP20 complex is involved in controlling expression of the nitrate transporter gene *NRT1.1* and the nitrate reductase genes *NIA1* and *NIA2* under conditions of N starvation [23]. It remains elusive, therefore, whether the PB1 domain is involved in regulating the expression of nitrate-activated genes.

**Fig. 1:**
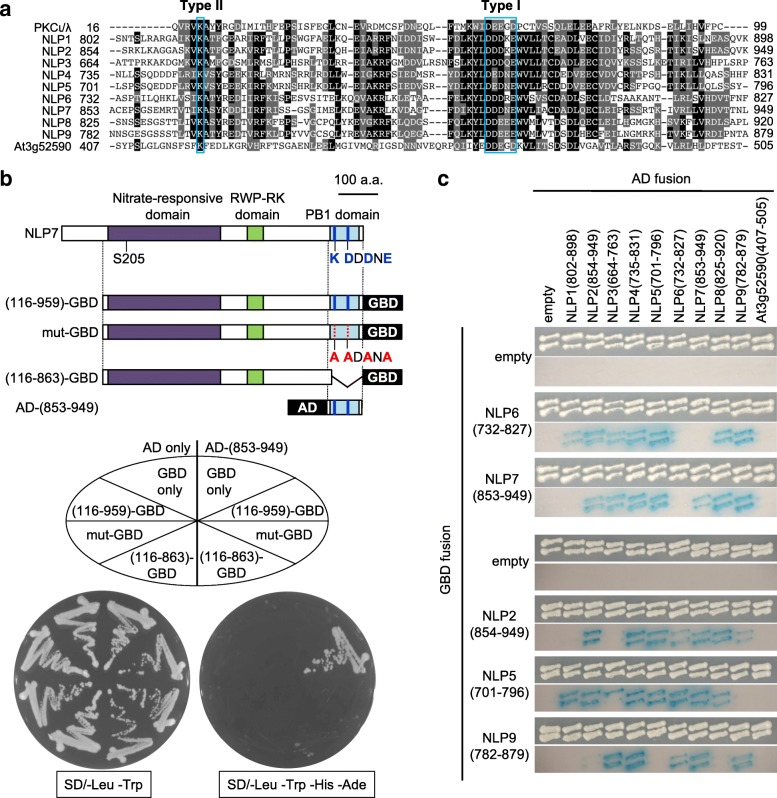
NLP-NLP interactions mediated by the PB1 domain in yeast. **a** Alignment of amino acid sequences of PB1 domains from Arabidopsis NLP transcription factors, human PKC1/λ, and an unrelated Arabidopsis protein (At3g52590). Black and gray backgrounds indicate identical and similar residues, respectively. Blue boxes mark core residues in Type I and Type II motifs. **b** Y2H assays with the PB1 domain of NLP7 (residues 853–949) as prey and wild-type NLP7 (amino acid residues 116–959), NLP7 containing mutations in the PB1 domain (K867, D909, D111, and E913 replaced with alanine residues), or NLP7 lacking the PB1 domain (residues 116–863) as bait. GBD and AD are the DNA-binding and transactivation domains of yeast Gal4, respectively; AD also contains a nuclear localization signal. **c** Y2H β-galactosidase assays to determine interactions between NLP PB1 domains. Numbers in parentheses indicate the amino acid residues used in the GBD and AD fusion constructs. For each GBD fusion construct, the upper and lower images show cell growth and β-galactosidase activity, respectively

The PB1 domain functions as a protein-protein interaction domain in a variety of proteins from animals, fungi, and plants; these include Cdc24 and Bem1, necessary for establishing cell polarity in budding yeasts, p40^phox^ and p67^phox^, which are involved in superperoxide formation in human phagocytes, and p62, which is required for autophagy in mammals and plants [31–34]. PB1 domains consist of about 80 amino acid residues and contain either or both the type I and type II motifs (Fig. 1a); they are thereby classified into type I, type II, and type I/II PB1 domains. The type I motif contains three glutamate or aspartate residues and occupies the back surface of the PB1 domain, whereas the type II motif contains an invariant lysine residue and is positioned in the front surface of the domain. The interaction between two PB1 domains thus occurs in a front-to-back manner, with electrostatic interactions between the basic lysine residue in one PB1 domain and the acidic glutamate/aspartate residues in the other [31]. Proteins containing type I PB1 domains hetero-dimerize with proteins containing cognate type II PB1 domains, whereas proteins containing type I/II PB1 domains may interact with type I, type II, and type I/II PB1 domains, and may also homo-dimerize [31, 32]. Furthermore, some PB1 domains mediate interactions with proteins lacking the PB1 domain [31]. NLP transcription factors possess a type I/II PB1 domain (Fig. 1a).

In this study, to identify proteins interacting with NLP transcription factors, we performed yeast two-hybrid (Y2H) screening with NLP7. We found that PB1 domain-mediated interactions occurred between a variety of NLP transcription factor combinations. Using a mutated form of NLP7 in which the amino acids necessary for PB1 domain-mediated protein-protein interactions were replaced with alanine, we showed that the PB1 domain was important for full activity of NLP7 *in planta*. However, disruption of the PB1 domain did not affect the ability of NLP7 to transactivate nitrate-inducible genes from plasmids in protoplast transient expression assays. Therefore, our results indicated that protein-protein interactions mediated by the PB1 domains of NLP transcription factors were necessary to fully promote expression of target genes in chromosomes but not to promote transcription from naked DNA. The effect reported here on nitrate-induced gene expression dependent on the PB1 domain differs from the NLP-TCP20 interaction, which occurs only under N-starvation conditions.

## Results

### PB1 domain-mediated interactions among NLP transcription factors

To identify proteins interacting with NLP transcription factors, we performed Y2H screening using NLP7 as bait. As the amino-terminal region of NLP7 was found to be a transcriptional activation domain (Additional file 1: Figure S1), we used NLP7 lacking the amino terminus (amino acids 116–959) to avoid artificial transactivation in yeast. We obtained multiple cDNA fragments that encompassed the PB1 domains of NLP2, NLP3, NLP4, NLP7, and NLP9, as well as cDNAs encoding chaperon-related proteins (Additional file 1: Figure S2); however, no cDNA encoding TCP20 was isolated in our screen. Protein-protein interactions were thus likely to involve a variety of combinations of NLP transcription factors.

To verify that NLP-NLP interactions depended on the PB1 domain, we first performed a Y2H assay using the wild-type and mutated forms of NLP7. To generate mutant NLP7 proteins, we either deleted the PB1 domain or replaced the core amino acid residues in the PB1 domain (K867, D909, D911, and E913) with alanine (Fig. 1b). The Y2H assay indicated that the native PB1 domain was necessary for the NLP-NLP interaction (Fig. 1b). Next, we repeated the Y2H assay using PB1 domains from other NLP transcription factors and the PB1 domain from an unrelated protein, At3g52590, as a negative control (Fig. 1a and c). This revealed that PB1 domain-mediated interactions occurred between a variety of NLP transcription factor combinations. We did not, however, detect the expected interaction between NLP6 and NLP7, nor observe interactions between some other combinations of proteins; this might be an experimental artefact caused by fusion of the PB1 domain to a domain of yeast Gal4. Alternatively, it might be due to a difficulty in co-expressing these proteins in yeast.

### Complementation of the *nlp6 nlp7–1* double mutant with NLP7 harboring mutations in the PB1 domain

**Fig. 2:**
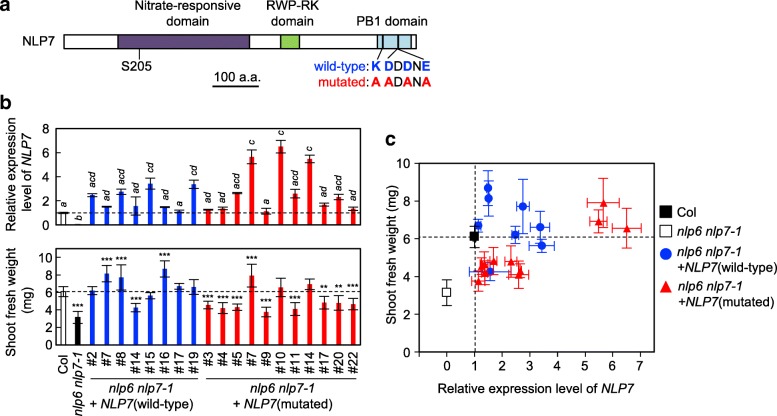
Complementation of the *nlp6 nlp7–1* double mutant by wild-type and mutant forms of NLP7. **a** The domain structure of the NLP7 protein. Core amino acid residues within the PB1 domain are labeled in blue font; these amino acid residues were replaced by alanine residues (red font) in the mutated protein. **b** Expression of introduced *NLP7* (upper panel) and shoot fresh weight (lower panel) of the transgenic lines. *nlp6 nlp7–1* double mutants were transformed with wild-type or mutated forms of the *NLP7* coding sequence under the control of the *NLP7* promoter. After germination, seedlings were grown on 0.5× MS agar plates for 4 days and then transferred to agar plates containing 10 mM KNO_3_ as a nitrogen source for 5 days. Col: wild-type Columbia seedlings. *NLP7* transcript levels were normalized against *UBIQUITIN10* (*UBQ10*) expression, and means ± SD (*n* = 3) are shown. Bars marked with different letters differ significantly from each other [Tukey’s honestly significant difference (HSD) test, *P* < 0.05]. Shoot fresh weight values are means ± SD (*n* = 9–10 plants). Asterisks indicate significant differences versus Col by one-way ANOVA with Dunnett’s post hoc test for multiple comparisons; **: *P* < 0.01 and ***: *P* < 0.001. **c** Correlations between *NLP7* expression and shoot fresh weight. Error bars are SD

To assess the role of the PB1 domain of NLP transcription factors *in planta*, we generated transgenic Arabidopsis expressing a mutated form of NLP7 in the *nlp6 nlp7–1* background. The double mutant, *nlp6 nlp7–1*, was used in this analysis as it displays stronger and more obvious phenotypes than the *nlp7–1* single mutant [35], making it easier to evaluate the effects of re-introducing functional *NLP7*. The mutated form of NLP7 contained a PB1 domain in which K867, D909, D111, and E913 were replaced with alanine residues (Fig. 2a), and the gene was expressed under the control of the *NLP7* promoter. Transgenic plants expressing wild-type *NLP7* from the *NLP7* promoter in the *nlp6 nlp7–1* background were generated as controls.

**Fig. 3:**
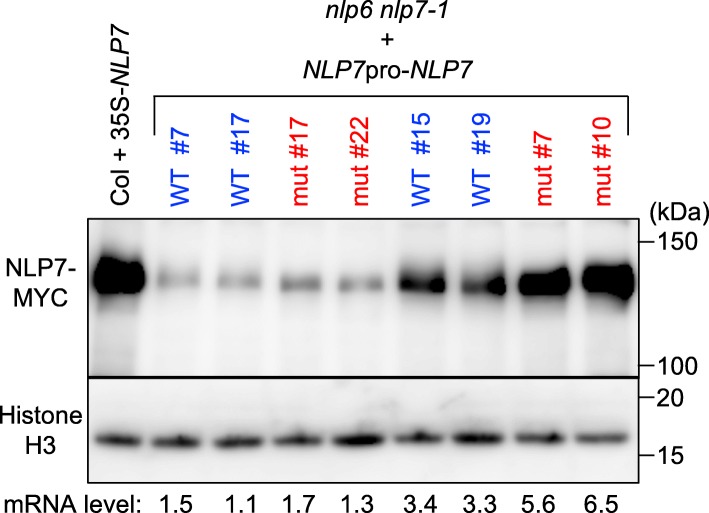
NLP7 protein levels in the complementation lines. WT and mut indicate transgenic lines expressing wild-type and mutated NLP7, respectively. After germination, seedlings were grown on 0.5× MS agar plates for 4 days and then transferred to agar plates containing 10 mM KNO_3_ as an N source for 5 days. NLP7 was detected using an anti-MYC antibody that recognizes the MYC tag at the carboxy terminus of the introduced NLP7. Histone H3 served as a loading control. The numbers below the lanes show the relative transcript levels of *NLP7* in these lines (Fig. 2b). A transgenic line over-expressing wild-type *NLP7* under the control of the *35S* promoter was used as a control for detection of the MYC tag

We first measured levels of *NLP7* transcript in multiple transgenic lines (Fig. 2b). Negligible levels of *NLP7* transcript were detected in *nlp6 nlp7–1* double mutants, indicating that *NLP7* transcript detected in the transgenic lines originated from the introduced *NLP7* gene. Transcript levels of *NLP7* varied between lines, probably because of positional effects. Levels of transcript in transgenic lines harboring wild-type *NLP7* were 1.1 to 3.4-fold those in wild-type Columbia (Col) plants, and all lines but one showed a full recovery in terms of shoot fresh weight. Expression of wild-type *NLP7* under the control of the native promoter fragment was thus sufficient to complement the loss of *NLP6* and *NLP7*. Only three of the transgenic lines harboring mutated *NLP7*, however, expressed high levels of *NLP7* transcript (5.4 to 6.5-fold that of Col); the same three lines showed a full recovery of shoot fresh weight, but the other eight lines, which expressed modest levels of *NLP7* transcript (1.2 to 2.6-fold that of Col), did not (Fig. 2b and c). To eliminate the possibility that mutant NLP7 was unstable, and therefore that higher levels of expression were required to rescue the *nlp6 nlp7–1* mutant, we used immunoblot analysis to examine NLP7 levels in these transgenic lines (Fig. 3). This analysis was performed using anti-MYC antibodies, as the NLP7 protein in the transgenic lines was tagged with MYC. The levels of detected protein in the transgenic lines were mostly in proportion with their *NLP7* mRNA expression, irrespective of whether they harbored wild-type or mutated protein. We therefore concluded that the mutated form of NLP7 did not possess the full activity needed to restore the growth of the *nlp6 nlp7–1* double mutant.

**Fig. 4:**
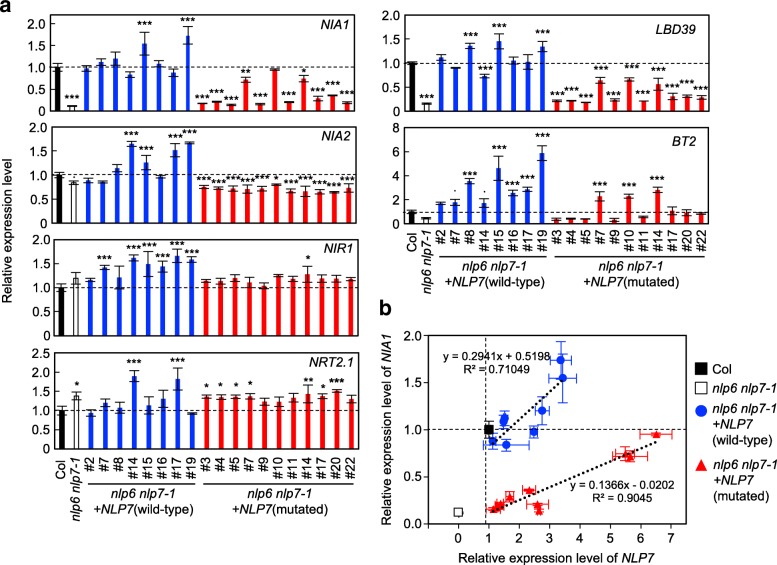
Effects of mutations in the PB1 domain on steady-state transcript levels from nitrate-inducible genes. **a** After germination, seedlings were grown on 0.5× MS agar plates for 4 days and then transferred to agar plates containing 10 mM KNO_3_ as a nitrogen source for 5 days. Transcript levels were normalized against *UBQ10* expression. Data are means ± SD (*n* = 3). Asterisks indicate significant differences versus Col by one-way ANOVA with Dunnett’s post hoc test for multiple comparisons; *: *P* < 0.05, **: *P* < 0.01, and ***: *P* < 0.001. **b** Correlations between *NLP7* and *NIA1* expression. Error bars are SD. The slopes for the wild-type and mutated *NLP7* differ significantly by analysis of covariance (*P* < 0.05)

Next, we compared steady-state levels of transcripts from NLP target genes, nitrate reductase genes *NIA1* and *NIA2*, *LBD39* (encoding a Lateral Organ Boundaries Domain (LBD) protein [4]), *BT2* (encoding a BTB-POZ domain protein [30]), *NIR1*, and *NRT2.1* (encoding a high affinity nitrate transporter). Transcript levels were determined in wild-type Col, *nlp6 nlp7–1*, and transgenic seedlings that had been grown for 9 days under nitrate-sufficient conditions (Fig. 4). Levels of *NIA1*, *LBD39*, and *BT2* transcripts were reduced in *nlp6 nlp7–1* seedlings, but limited effects on steady-state levels of *NIA2*, *NIR1*, and *NRT2.1* transcripts were observed (Fig. 4a). Expression of *NIA1*, *LBD39*, and *BT2* was restored in the transgenic lines expressing wild-type NLP7. Expression of *NIA1* and *LBD39* was also restored in the three transgenic lines that expressed high levels of mutated *NLP7* transcript (lines #7, #10, and #14) but not in the other eight lines, which expressed only modest levels of mutated *NLP7* transcript (Fig. 4a). Transcript levels of *BT2* also showed similar pattern (Fig. 4a). The gradients of the regression lines for the correlation between the *NLP7* and *NIA1* transcript levels were evidently different for the transgenic lines expressing the wild-type and mutated NLP7 (Fig. 4b). The line of best fit obtained from the regression analysis was steeper in the lines expressing wild-type NLP7 than in the lines expressing mutated protein, indicating that the activity of mutated NLP7 was severely compromised.

### Effects of mutations in the PB1 domain on nitrate-inducible gene expression *in planta*

**Fig. 5:**
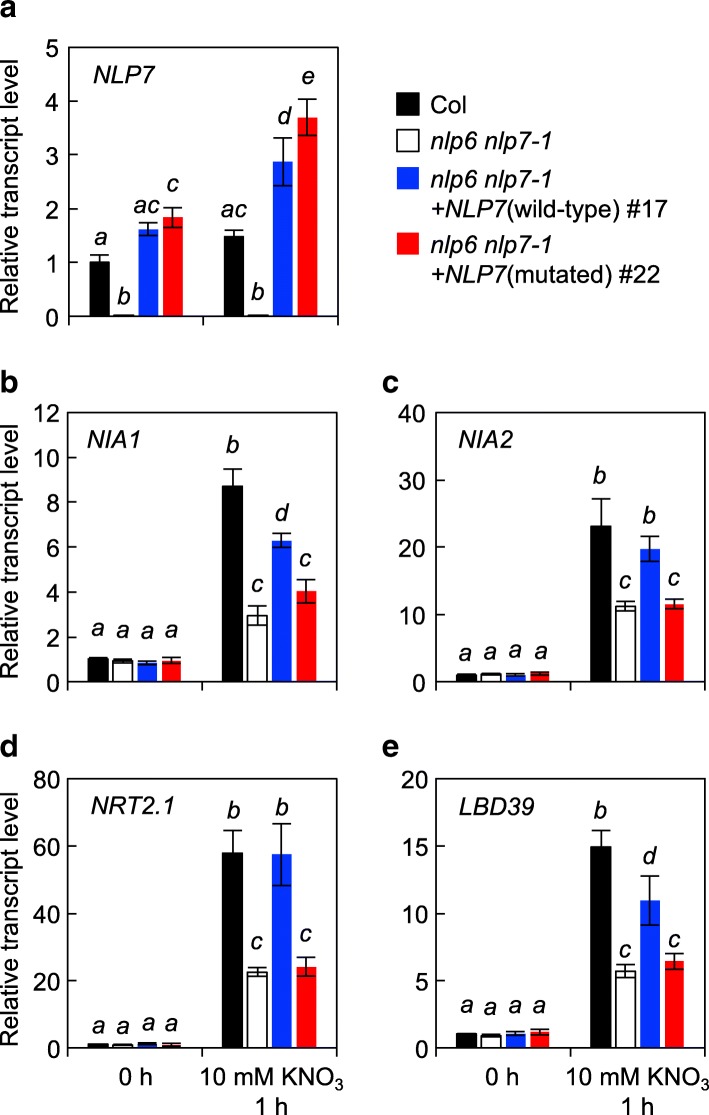
Effects of mutations in the PB1 domain on nitrate-induced expression. Expression levels of *NLP7* (**a**) and four nitrate-inducible genes, *NIA1* (**b**), *NIA2* (**c**) *NRT2.1* (**d**) and *LBD39* (**e**) were analyzed. Seedlings of Col, *nlp6 nlp7-1*, and the complementation lines expressing wild-type or mutated NLP7 were grown with ammonium as the sole N source and then treated with 10 mM nitrate for 1 h. Transcript levels were normalized against *UBQ10* expression; means ± SD (*n* = 3) are shown. Bars marked with different letters differ significantly from each other (Tukey’s HSD test, *P* < 0.05)

To evaluate the ability of mutated NLP7 to activate nitrate-inducible gene expression, we used qRT-PCR to analyze transcript levels in seedlings grown in nitrate-free conditions and 1 h after the addition of nitrate. A pair of transgenic lines expressing comparable levels of *NLP7* transcript to Col seedlings was selected from the lines harboring wild-type and mutated forms of NLP7 (Fig. 5a). Addition of nitrate induced expression of *NIA1*, *NIA2*, *NRT2.1*, and *LBD39* in *nlp6 nlp7–1* seedlings to approximately half the levels seen in Col seedlings (Fig. 5b-e). This difference in expression almost disappeared in *nlp6 nlp7–1* seedlings expressing wild-type NLP7; by contrast, expression levels of these genes after nitrate application were similar in the *nlp6 nlp7–1* seedlings and the transgenic line expressing mutant NLP7 (Fig. 5b-e). Very similar results were obtained from a second, independent pair of transgenic lines (Additional file 1: Figure S3). Addition of nitrate increased levels of both wild-type and mutated *NLP7* transcripts to only a limited extent (Fig. 5a). Differences in nitrate-inducible gene expression did not therefore result from an artificial reduction in the level of mutated NLP7 transcript caused by nitrate treatment. Mutated NLP7 thus failed to promote expression of nitrate-inducible genes. It should be noted that expression of *NRT2.1* was lower in *nlp6 nlp7–1* double mutants than in Col seedlings under conditions designed to monitor short-term responses to nitrate (Fig. 5d), whereas a reduction in *NRT2.1* expression was not apparent in *nlp6 nlp7–1* mutants grown with a continuous supply of nitrate (Fig. 4a). Long-term incubation with nitrate may result in downregulation of nitrate signaling and an increase in N metabolites, both of which mask the direct effect of the *nlp* mutations.

### Effects of mutations in the PB1 domain on transactivation of NLP7 in protoplast transient expression assays

To investigate the role of the PB1 domain in the transactivation of target genes, we performed protoplast transient expression assays. We first confirmed that the PB1 domain alone was unable to induce transcriptional activation (Additional file 1: Figure S1d), and then evaluated the ability of wild-type NLP7 and a mutant form of NLP7 carrying amino acid substitutions in the PB1 domain to transactivate target genes.

**Fig. 6:**
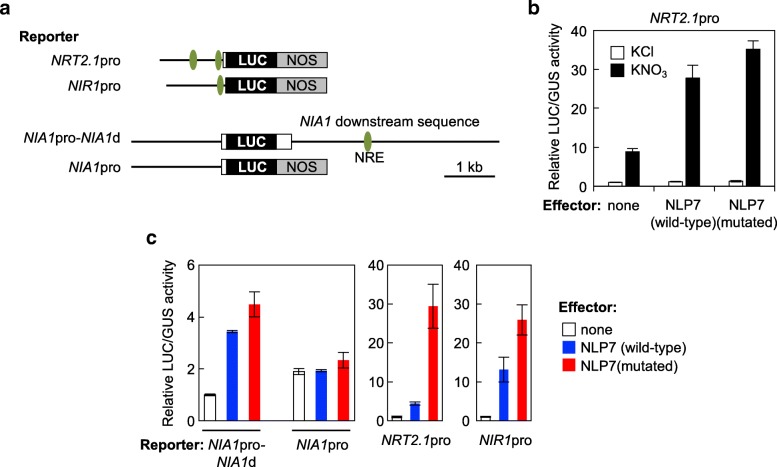
Activity of mutated NLP7 in a protoplast transient assay system. **a** Schematic representation of the reporter constructs used in **b** and **c**; LUC: firefly luciferase gene; NOS: transcription termination sequence of the nopaline synthase gene. White boxes indicate 5′ or 3′ untranslated regions, and horizontal lines indicate sequences upstream (“promoter”) or downstream of the *NRT2.1*, *NIR1*, or *NIA1* coding regions. Green ovals mark experimentally verified NLP-binding sites. **b** Protoplast transient assay using N-starved Col protoplasts. Protoplasts were co-transfected with the *NRT2.1*pro reporter plasmid, effector plasmids for expression of wild-type or mutated NLP7, and a control plasmid expressing β-glucuronidase (GUS) under the control of the *UBQ10* promoter (UBQ10-GUS) and incubated overnight in medium supplemented with either 1 mM KCl or KNO_3_. **c** Protoplast transient assay using protoplasts isolated from leaves of *nlp6 nlp7–1* plants supplied with nitrogen. Protoplasts were co-transfected with the reporter plasmid, plasmids for expression of NLP7, and the control plasmid, and incubated overnight in protoplast incubation medium. Luciferase activity values were normalized against GUS activity. Means ± SD (*n* = 3) are shown in **b** and **c**

Plasmids containing wild-type or mutant *NLP7* under the control of the *35S* constitutive promoter were introduced into mesophyll protoplasts isolated from nitrogen-starved Col plants, together with a plasmid containing the *LUC* reporter under the control of the *NRT2.1* promoter (Fig. 6a). Transfected protoplasts were incubated in the presence or absence of nitrate. The wild-type and mutated forms of NLP7 enhanced nitrate-dependent activation of the *NRT2.1* promoter to a similar extent (Fig. 6b). This was an unexpected result, as it implied that protein-protein interactions mediated by the PB1 domain were not necessary for transactivation of target promoters in protoplasts.

All the *in planta* analyses shown in Figs. 2, 3, 4 and 5 involved *nlp6 nlp7–1* plants expressing wild-type or mutant NLP7. To understand the discrepancy between the results shown in Figs. 5 and 6b, we performed further transient assays using protoplasts isolated from leaves of *nlp6 nlp7–1* plants grown under N-sufficient conditions. Mutant NLP7 protein strongly activated expression of the *LUC* reporter under the control of the *NIR1* and *NRT2.1* promoters in *nlp6 nlp7–1* protoplasts, and the increase in *LUC* activity associated with the mutant NLP7 protein was slightly higher than that associated with wild-type NLP7 (Fig. 6c). Mutant NLP7 also promoted expression of the *LUC* reporter via an interaction with the NLP-binding site located downstream of *NIA1* (Fig. 6c). As the NRE in *NIA1* is positioned in the 3′ flanking region downstream of *NIA1* [13, 36, 37], NLP7 (both wild-type and mutant forms) activated the NIA1pro-NIA1d reporter but not the NIA1pro reporter (Fig. 6c). All these results indicate that mutant NLP7 protein itself retains DNA-binding and transactivation activities.

## Discussion

We isolated cDNA clones encoding various NLP transcription factors in a Y2H screen designed to identify proteins interacting with NLP7. Furthermore, we showed that most of the PB1 domains found in NLP transcription factors interacted with each other in a variety of combinations (Fig. 1c). This result strongly suggested that, as well as the previously reported interactions between NLP6 and NLP6, NLP6 and NLP7, and NLP7 and NLP7 [23], interactions involving other combinations of NLP transcription factors with overlapping expression patterns are likely to occur in plant cells. Interactions between NLP transcription factors are canonical PB1 domain-mediated interactions, as they are abolished by mutations in core amino acid residues in the PB1 domain (Fig. 1b). Our Y2H analysis did not, however, detect the interactions between NLP6 and NLP6 or between NLP6 and NLP7 that were identified previously using tobacco cells [23], possibly because an experimental artefact was caused by fusion of the PB1 domain to a domain of yeast Gal4 or because expression levels of these proteins in yeast were too low.

Our complementation experiments revealed that mutations in the PB1 domain strongly compromised NLP7-dependent activation of nitrate-inducible genes in response to nitrate (Fig. 5; Additional file 1: Figure S3). We also showed that the PB1 domain was necessary for steady-state levels of expression of some nitrate-inducible genes (Fig. 4). These results provide direct evidence that interaction(s) mediated by the PB1 domain are required for full NLP7 function in the presence of nitrate. Previously, because of the correlation between the NLP6/7-TCP20 interaction, which is dependent on N starvation, and the phenotype of the *tcp20* single mutant, the interaction between NLP and TCP20 was considered to regulate gene expression only under N-starvation conditions [23]. We found, however, that nitrate-inducible genes were activated in a PB1 domain-dependent manner under nitrate-sufficient conditions, and thus this regulation by the PB1 domain should be distinguishable from regulation dependent on the NLP-TCP20 interaction.

Unexpectedly and interestingly, similar levels of transcription were induced by wild-type and mutant NLP7 in protoplast transient assays (Fig. 6), indicating that mutant NLP7 could be activated by nitrate signaling, bind to DNA, and activate transcription at a level comparable to wild-type NLP7, and thus retained completely the ability to act as a transcription factor. Despite this finding, *in planta* gene expression in the transgenic lines was differentially regulated by wild-type and mutated forms of NLP7 (Figs. 4 and 5). Although the protoplast transient assay system is a powerful tool for evaluating the effects of transcription factors on target promoters [38], it does not completely mimic transcription of chromosomal genes. DNA in reporter plasmids transiently introduced into protoplasts is in a naked state, whereas chromosomal DNA is wrapped around histones, forming nucleosomes. As nucleosomes serve as a general barrier against transcription [39], oligomerization of NLP proteins mediated by their PB1 domains might be required to induce chromatin remodeling, thus enabling transcription.

Recently, a PB1-mediated interaction of *Medicago truncatula* NLP1 with a nodule-specific transcription factor, NODULE INCEPTION (NIN), was reported in *M. truncatula* [17]. As their full name “NIN-LIKE PROTEIN” suggests, proteins from the NLP family show considerable similarities to NIN. Leguminous NIN proteins contain RWP-RK DNA-binding and PB1 domains, as well as a region resembling the nitrate-responsive region found in NLP transcription factors. *Lotus japonicus* NIN functions as a transcriptional activator with a similar DNA-binding specificity to NLP transcription factors, but *Lj*NIN has lost the ability to respond to nitrate due to mutations in its nitrate-responsive region [19, 26]. PB1-mediated interactions between NIN and NLP transcription factors are therefore very plausible. The interaction between *Mt*NLP1 and *Mt*NIN represses NIN activity, and thus inhibits induction of nodulation in response to nitrate [17]. This suggests that interactions mediated by the PB1 domain may have a positive effect on NLP transcription factors but exert a repressive effect on NIN activity. Further analyses are therefore necessary to understand exactly how NLP-NLP and NLP-NIN interactions produce different outputs.

## Conclusion

The PB1 domain of NLP transcription factors mediated a variety of interactions between different NLP transcription factors. Protein-protein interactions mediated by the PB1 domain were necessary for the full expression of nitrate-induced genes *in planta*. Such interactions may recruit chromatin remodeling factors through self-oligomerization of NLP transcription factors mediated by the PB1 domain. Further analyses of these protein-protein interaction(s) may provide clues leading to a full understanding of the NLP/NIN network that regulates nitrate-linked physiological processes.

## Methods

### Plasmid construction

For Y2H analysis, pGBT9K-MCS-GBD that enables to express carboxy-terminal Gal4 DNA-binding domain (GBD) fusion proteins was generated using pGBT9 (Clontech, Mountain View, CA, USA), a plasmid vector enabling the production of amino-terminal GBD fusion proteins. The *Hin*dIII site between the *ADH1* promoter and the GBD coding sequence in pGBT9 was replaced with a sequence containing *Bam*H1, *Nco*I and *Stu*I sites, and a multiple-cloning site (MCS) between the GBD sequence and the *ADH1* terminator was removed. To generate pNLP7(116–959)-GBD and pNLP7(116–863)-GBD for Y2H analysis, the DNA fragments encompassing amino acids 116–959 or 116–863 of NLP7 were amplified by PCR using appropriate PCR primers attached with either an *Nco*I or *Stu*I site. Then, the PCR products were cloned into pGBT9K-MCS-GBD using the *Nco*I and *Stu*I sites between the *ADH1* promoter and the GBD coding sequence. To generate the plasmid pNLP7(PB1mut)-GBD, mutations were introduced into the PB1 domain by five rounds of PCR using mutated primer sets (Additional file 1: Table S1). The mutated fragment was also inserted between the *Nco*I and *Stu*I sites in pGBT9K-MCS-GBD. To obtain plasmids for analyzing interactions between PB1 domains, DNA fragments encompassing the PB1 domain were amplified by PCR with appropriate PCR primers attached with either an *Eco*RI or *Bam*HI, and the resultant PCR products were inserted into pGBT9 or pGADT7 (Clontech).

The reporter plasmids, pNRT2.1pro-LUC [40] and pNIR1pro-LUC [13], and the effector plasmid p35SC4PPDK-NLP7-MYC6 [13] used in co-transfection assays have been described previously. The plasmid pNIA1pro-LUC-NOS was generated by replacing the *35S* promoter in pJD301 [41] with a 1.9 kb DNA fragment from the *NIA1* promoter [36]. pNIApro-LUC-NIA1d was generated by replacing the *NOS* terminator region in pNIA1pro-LUC-NOS with the 4.5 kb DNA fragment downstream of the stop codon of *NIA1* [36]. To produce effector plasmids enabling expression of a particular region of NLP7 fused to LexA, the MYC tag coding sequence in the LexA-MYC plasmid was replaced with DNA fragments encoding particular regions of NLP7, which were obtained by PCR amplification [42]. All the primers used are listed in Additional file 1: Table S1. All the cloned fragments obtained by PCR amplification were confirmed by DNA sequencing.

### Yeast transformation and β-galactosidase assay

*Saccharomyces cerevisiae* strains AH109 and Y187 were used for the growth assay and the β-galactosidase assay, respectively. Yeast was transformed with plasmids based on pGBT9 and pGADT7 using lithium acetate/polyethyleneglycol-mediated transformation, and transformed colonies were selected on Synthetic Defined (SD) medium lacking tryptophan and leucine. For the growth assay, colonies were streaked onto SD medium lacking tryptophan, leucine, histidine, and adenine. For the β-galactosidase assay, colonies were streaked onto SD medium lacking tryptophan and leucine, and a colony-lift filter assay was performed after 2 days of incubation, following the methodology of the Yeast Protocols Handbook [43].

### Plant materials and growth conditions

*Arabidopsis thaliana* ecotype Columbia (Col-0) derived from the Arabidopsis Biological Resource Center (ABRC) was maintained in-house and used as the wild-type control in this study. The *nlp6* and *nlp7–1* mutants were also obtained from the ABRC and crossed to generate the *nlp6 nlp7–1* double mutant [40]. Arabidopsis plants for propagation were grown on a nutrient-containing peat (Jiffy-7, Sakatanotane, Yokohama, Japan) and irrigated with water. The *nlp6 nlp7–1* mutant was irrigated with water containing 1 mM CaCl_2_.

For the measurements of shoot fresh weight, and determinations of transcript and protein levels, seeds were sown on plates containing 0.5× MS [half-strength Murashige and Skoog (MS) salts [44], 0.5 g L^− 1^ 2-morpholinoethanesulfonic acid, monohydrate (MES)-KOH, pH 5.7, 1% sucrose, and 0.8% agar (Sigma A1296)] and stratified at 4 °C for 3–4 days. Plates were placed horizontally in a growth chamber set at 23 °C with continuous illumination (60 μmol m^− 2^ s^− 1^) for 4 days. Seedlings were then transferred to vertical test plates (nitrogen-free 0.5× MS salts, 10 mM KNO_3_, 0.5 g L^− 1^ MES-KOH, pH 5.7, 1% sucrose, and 0.8% agar) and incubated for 5 days. To avoid direct contact between shoots and the medium [45], the agar medium was removed from the upper 2 cm of the test plates before incubation. To analyze expression of nitrate-induced genes, 100 seeds were surface-sterilized and sown in 20 ml liquid ammonium medium (nitrogen-free 0.1× MS salts, 0.1 g L^− 1^ MES-KOH, pH 5.7, 0.5% sucrose, and 0.5 mM ammonium succinate) in a plastic dish. After stratification at 4 °C for 3–4 days, the dishes were transferred to 23 °C under continuous light (60 μmol m^− 2^ s^− 1^). After 4 days, seedlings were treated with 10 mM KNO_3_ for 1 h before sample collection, followed by RNA extraction and RT-qPCR.

### Binary plasmid construction and generation of transgenic plants

A 3 kb DNA fragment of the *NLP7* promoter (base pairs − 3018 to − 1, relative to the translation start site) was amplified by PCR, digested with *Hin*dIII and *Nco*I, and cloned into pJD301 to generate the plasmid pNLP7pro-LUC. All the primers used are listed in Additional file 1: Table S1. The promoter fragment was then excised from pNLP7pro-LUC, and the *NLP7* coding sequence, together with the sequences encoding six copies of the MYC tag and the *NOS* terminator, was excised from p35SC4PPDK-NLP7-MYC6 [13]. The two sequences were inserted together into pCB302HYG-35SΩ-GUS [46] to replace the 35SΩ promoter, the *GUS* gene, and the *NOS* terminator. The resulting binary plasmid was introduced into *Agrobacterium tumefaciens* strain GV3101 (pMP90) [47], which was used to transform the *nlp6 nlp7–1* double mutant. T2 generation lines showing 3:1 segregation for hygromycin resistance were selected, and used to generate homozygous lines, selected at the T3 generation.

### RT-qPCR

RNA extraction, reverse transcription, and quantitative PCR (qPCR) were performed as described previously [28]. Primers used in qPCR analyses are listed in Additional file 1: Table S1.

### Protoplast co-transfection assay

The protoplast co-transfection assay using hydroponically grown, N-starved Col plants was performed as previously described [40], with the modification that 0.1 μg of the effector plasmid was added per 2 × 10^4^ protoplasts in this study. *nlp6 nlp7–1* seeds were placed on a urethane sponge moistened with 0.1× MS solution supplemented with 2 mM CaCl_2_ (0.1× MS salts, 0.1 g L^− 1^ MES-KOH, pH 5.7, 2 mM CaCl_2_), and incubated under continuous illumination for 1 day and then in the dark for 2 days. Etiolated *nlp6 nlp7–1* seedlings were then grown hydroponically in 0.1× MS solution supplemented with 2 mM CaCl_2_ in the light for 20 days. Protoplast isolation and transfection of 2 × 10^4^ protoplasts with reporter plasmid (6 μg), effector plasmid (0.1 μg), the internal control plasmid UBQ10-GUS (2 μg), and the empty plasmid (12 μg) were all as described previously. Transfected protoplasts were incubated in WI medium overnight in the dark.

### Immunoblot analysis

Sample preparation, SDS-PAGE, and immunoblot analysis were all performed as described in Liu et al. [15].

## Additional file

Additional file 1: **Figure S1.** The non-conserved amino-terminal region of NLP7 is a transactivation domain. **Figure S2.** Clones obtained in a Y2H screen with NLP7 (aa. 116–959) as bait. **Figure S3.** Effects of mutations in the PB1 domain on nitrate-induced gene expression. **Table S1.** List of primers used in the study. (PDF 66 kb)

## Abbreviations

AD: Gal4 transactivation domain
GBD: Gal4 DNA-binding domain
GUS: β-glucuronidase
LBD: Lateral Organ Boundaries Domain
MCS: Multiple-cloning site
N: Nitrogen
NLP: NIN-LIKE PROTEIN
NOS terminator: Transcription termination sequence of nopaline synthase
NRE: Nitrate-responsive *cis*-element
PB1: Phox and Bem1
Y2H: Yeast two-hybrid
