# Supplementary material for: The role of protein-protein interactions mediated by the PB1 domain of NLP transcription factors in nitrate-inducible gene expression
Source: BMC Plant Biol. 2019 Feb 28;19:90. doi: 10.1186/s12870-019-1692-3 (PMC6393987; doi:10.1186/s12870-019-1692-3)
Supplement: Supplementary file 1 — Figure S1. The non-conserved amino-terminal region of NLP7 is a transactivation domain. Figure S2. Clones obtained in a Y2H screen with NLP7 (aa. 116–959) as bait. Figure S3. Effects of mutations in the PB1 domain on nitrate-induced gene expression. Table S1. List of primers used in the study. (PDF 66 kb) [file 12870_2019_1692_MOESM1_ESM.pdf]

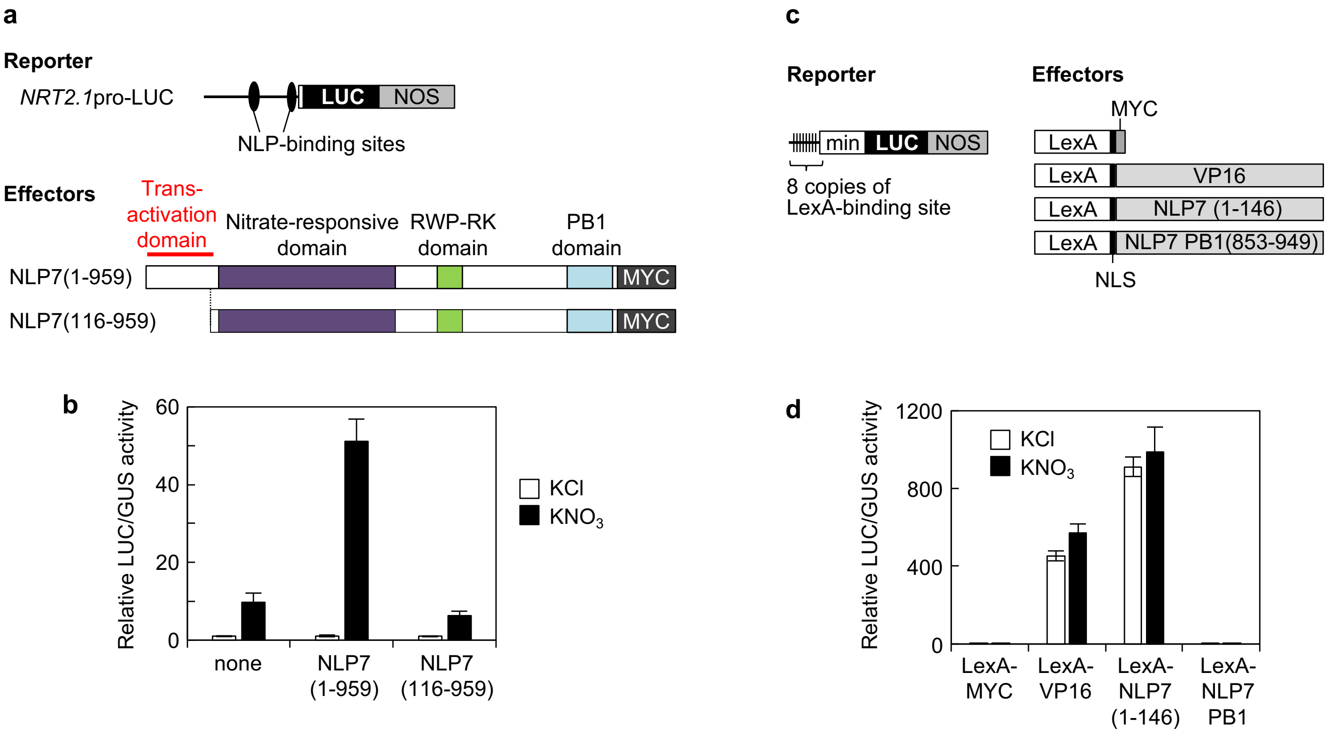

Figure S1. The non-conserved amino-terminal region of NLP7 is a transactivation domain. **a** Schematic representation of the reporter and effector constructs used in **b**. The white box indicates the 5' untranslated region, and the horizontal line indicates the *NRT2.1* promoter. Black ovals mark experimentally verified NLP-binding sites. LUC: luciferase gene; NOS: transcription termination sequence of the nopaline synthase gene. The full-length NLP7 (amino acids 1–959) or NLP7 truncated at the amino terminus (amino acids 116–959) was used as an effector. MYC: MYC tag. **b** Deletion of 115 amino acids from the amino terminus of NLP7 resulted in the loss of transactivation. Nitrogen-starved Col protoplasts were co-transfected with the *NRT2.1*pro reporter plasmid, effector plasmids for expression of full-length or truncated NLP7, and a control plasmid expressing β-glucuronidase (GUS) under the control of the *UBQ10* promoter (UBQ10-GUS), and incubated overnight in medium supplemented with either 1 mM KCl or KNO_3. **c** Schematic representation of the reporter and effector constructs used in **d**. min: minimal promoter derived from the *35S* promoter; LexA: a bacterial DNA-binding protein; NLS: nuclear localization signal to ensure nuclear localization of LexA; MYC: MYC tag; VP16: a viral transactivation domain used as a positive control. **d** Assessment of transcriptional activation activity of amino acids 1–146 and the PB1 domain in a protoplast transient assay. N-starved Col protoplasts were co-transfected with the LexA reporter plasmid and effector plasmids for expression of LexA fusion proteins and UBQ10-GUS and incubated overnight in medium supplemented with either 1 mM KCl or KNO_3. LUC activities were normalized against those of GUS. Means ± SD are shown (n = 3).

a

NLP:
- *NLP2*, 1 clones
- *NLP3*, 7 clones
- *NLP4*, 1 clone
- *NLP7*, 5 clones
- *NLP9*, 1 clone

Chaperone-related:
- *ATJ3* (*DNA J homologue 3*), 5 clones
- *TPR10*, 1 clone

b

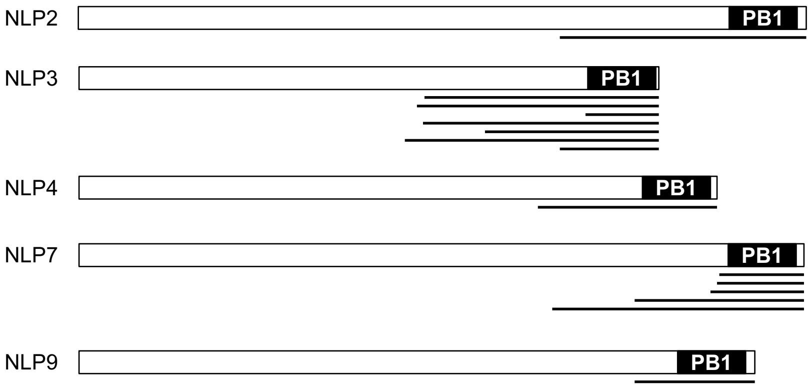

Figure S2. Clones obtained from Y2H screening using amino acids 116–959 from NLP7 as a bait. **a** List of clones obtained in Y2H screening. **b** The positions of the regions encoded by the cDNA clones isolated relative to representations of the NLP proteins.

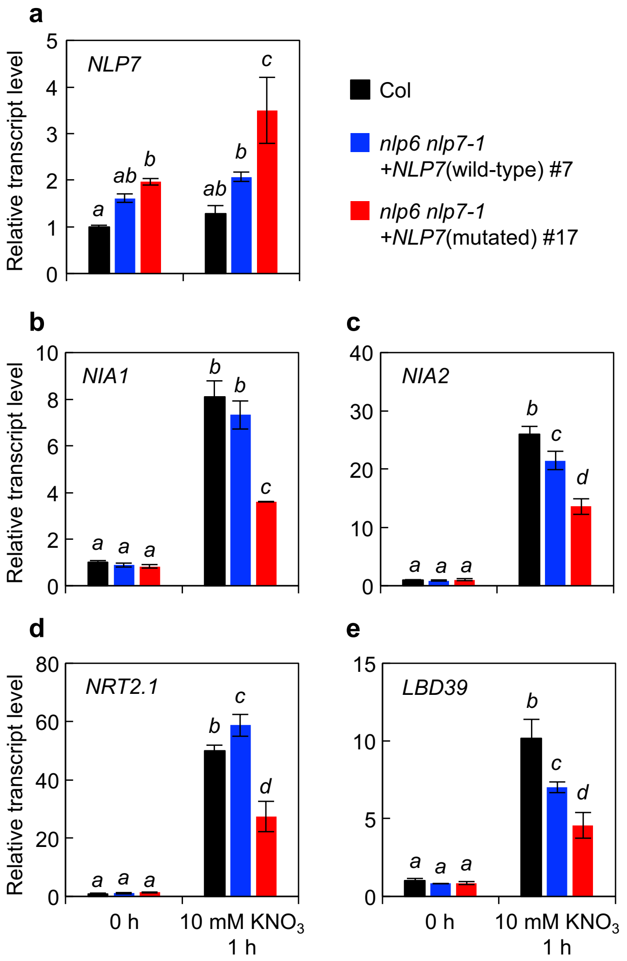

Figure S3. Effects of mutations in the PB1 domain on nitrate-induced expression of *NIA1*, *NIA2*, *NRT2.1*, and *LBD39*. Seedlings of Col and the complementation lines harboring wild-type or mutated NLP7 were grown with ammonium as the sole N source and then treated with 10 mM nitrate for 1 hour. Transcript levels were normalized against *UBQ10* expression. Means ± SD (n = 3) are shown. Bars marked with different letters differ significantly from each other (Tukey's HSD test, $P < 0.05$).

Table S1. List of primers used in the study

Amplicon	Gene	AGI code	Sequence
cloning for Y2H			
Gal4 DNA-binding domain (GBD)			GTTAGGCCTATGAAGCTACTGTCTTCTATCGAAC<br>GTTCTGCAGCGATACAGTCAACTGTCTTTGACC
NLP7 (aa.116-959)	*NLP7*	AT4G24020	GTGCCATGGCTGAGAACACAACAGAGAAGCAT<br>GTCAGGCCTCAATTCTCCAGTGCTCTCGCAGGA
NLP7 (aa. 116-863)	*NLP7*	AT4G24020	GTGCCATGGCTGAGAACACAACAGAGAAGCAT<br>GTGAGGCCTCGTTCTCATTTCTGAGCCTGATGG
NLP7 PB1 domain mutation PCR1	*NLP7*	AT4G24020	GTTGAATTCGCCACTGTTAACGGTGTGGTTAAG<br>TAACTTGCTGCGATTGTTACCGTTCTCATTTC
NLP7 PB1 domain mutation PCR2	*NLP7*	AT4G24020	GTAACAATCGCAGCAAGTTACAAAGACGACA<br>CCATGCGTTAGCATCGGCAAGATACTTGATATCGAACG
NLP7 PB1 domain mutation PCR3	*NLP7*	AT4G24020	TTGCCGATGCTAACGCATGGGTTTTAATAGCTTGTGATG<br>GTCAGGCCTCAATTCTCCAGTGCTCTCGCAGGA
NLP7 PB1 domain mutation PCR4<br>(using PCR2 + PCR3 as template)	*NLP7*	AT4G24020	GTAACAATCGCAGCAAGTTACAAAGACGACA<br>GTCAGGCCTCAATTCTCCAGTGCTCTCGCAGGA
NLP7 PB1 domain mutation PCR5<br>(using PCR4 + PCR1 as template)	*NLP7*	AT4G24020	GTTGAATTCGCCACTGTTAACGGTGTGGTTAAG<br>GTCAGGCCTCAATTCTCCAGTGCTCTCGCAGGA
NLP1PB1 (aa. 802-898)	*NLP1*	AT2G17150	GTCGAATTCAGCAACACGAGTTTAAGAGCTAGA<br>GTCGGATCCTCACTTGACTTGAGAAGCTTCGTTCAG
NLP2PB1 (aa. 854-949)	*NLP2*	AT4G35270	GTCGAATTCAGCAGGAAGTTGAAAGCTGGAG<br>GTCGGATCCTTACTTGACTTGAGAAGCTTCATGAAC
NLP3PB1 (aa. 664-763)	*NLP3*	AT4G38340	GTCGAATTCGCAACTACTCCAAGGAAAGCAAAG<br>GTCGGATCCTTAAGGACGAGAAAGAGGATGGTGAAC
NLP4PB1 (aa. 735-831)	*NLP4*	AT1G20640	GTCGAATTCAACTTGTTATCATCTCAAGATGATGA<br>GTCGGATCCTCAGAAATGATGAGAAGAAGCCTGAAG
NLP5PB1 (aa. 701-796)	*NLP5*	AT1G76350	GTCGAATTCTTATCACCATCATCACAAGAGGAT<br>GTCGGATCCTCAATAAGAAGAAGAGAGCTGAAGCAA
NLP6PB1 (aa. 732-827)	*NLP6*	AT1G64530	GTCGAATTCGCTTCTCCAACAATTCTCCAACAT<br>GTCGGATCCTCAAAAGTTAAAAGTCACGTCATGTAC
NLP7PB1 (aa. 853-949)	*NLP7*	AT4G24020	GTCGAATTCGCATGCGAACCATCAGGCTCAG<br>GTCGGATCCTCATAGATTTGTCGTTACATCATGAAC
NLP8PB1 (aa. 825-920)	*NLP8*	AT2G43500	GTCGAATTCAGTAATAGCAGCGAGAGTGGATC<br>GTCGGATCCTCATAGAGGGGCAGACAAATCACGAAC
NLP9PB1 (aa. 782-879)	*NLP9*	AT3G59580	GTCGAATTCAACAATAGCGGTGAAAGCGGATCA<br>GTCGGATCCTTATGCGGTGTTCGGTATATCACGG
AT3G52950PB1 (aa. 407-505)	AT3G52950	AT3G52950	GTCGAATTCTCTTACCCATCTCTAGGACTAGG<br>GTCGGATCCTCATGTTGACTCAGTGAAGTCCAGATG
RT-qPCR			
NLP7	*NLP7*	AT4G24020	TGTATCTGCAGCTTCCTTCG<br>TTGAACTTCCAGCGTCTTCA
NIA1	*NIA1*	AT1G77760	GAAATCGCAAAGGAAGGTTG<br>ACTGAATCATAGGCGGTGGT
NIA2	*NIA2*	AT1G37130	AAGGGAGGAACTGGATGGTT<br>CGGTACTGTATGCCCAACCT
NIR1	*NIR1*	AT2G15620	CATGGGATGCTTAACACGAG<br>AATGGAACCAACTCCGTGAC
NRT2.1	*NRT2.1*	AT1G08090	TGAGCAGGAGAAGCAGAAGA<br>TTGTTGGGTGTGTTCTCAGG
LBD39	*LBD39*	AT4G37540	CAAGAAACCAAACCCACCAT<br>CGTGGTTCACTTGGAGATCA
BT2	*BT2*	AT3G48360	TCCATTCGCAGTTTAAGACC<br>AACTGGAGAATGTCGAGCTC
UBQ10	*UBQ10*	AT4G05320	GGCCTTGTATAATCCCTGATGAATAAG<br>AAAGAGATAACAGGAACGGAAACATAGT
Cloining of the NLP7 promoter for binary plasmid			
NLP7 promoter	*NLP7*	AT4G24020	GTGAAGCTTAGGAAGAGTTAGAACGTTAATGGC<br>GAGCCATGGATCCAAAGCAGTTTCTGGAATTTTC
Cloning for LexA fusion			
NLP7(aa. 1-146)	*NLP7*	AT4G24020	GTCAGGCCTATGTGCGAGCCCGATGATAATTCC<br>CATCTGCAGTCACACACAGTAGTTGTCTGTGTTC
NLP7PB1 (aa. 853-949)	*NLP7*	AT4G24020	GTCAGGCCTGCATGCGAACCATCAGGCTCAG<br>GTCCTGCAGTCATAGATTTGTCGTTACATCATGAAC
